# Keeping It Organized: Multicompartment Constructs to Mimic Tissue Heterogeneity

**DOI:** 10.1002/adhm.202202110

**Published:** 2023-04-02

**Authors:** Alvaro Sanchez‐Rubio, Vineetha Jayawarna, Emily Maxwell, Matthew J. Dalby, Manuel Salmeron‐Sanchez

**Affiliations:** ^1^ Centre for the Cellular Microenvironment University of Glasgow Glasgow G11 6EW UK

**Keywords:** 3D bioprinting, hydrogels, in vitro models, multicompartment models, tissue engineering

## Abstract

Tissue engineering aims at replicating tissues and organs to develop applications in vivo and in vitro. In vivo, by engineering artificial constructs using functional materials and cells to provide both physiological form and function. In vitro, by engineering three‐dimensional (3D) models to support drug discovery and enable understanding of fundamental biology. 3D culture constructs mimic cell–cell and cell–matrix interactions and use biomaterials seeking to increase the resemblance of engineered tissues with its in vivo homologues. Native tissues, however, include complex architectures, with compartmentalized regions of different properties containing different types of cells that can be captured by multicompartment constructs. Recent advances in fabrication technologies, such as micropatterning, microfluidics or 3D bioprinting, have enabled compartmentalized structures with defined compositions and properties that are essential in creating 3D cell‐laden multiphasic complex architectures. This review focuses on advances in engineered multicompartment constructs that mimic tissue heterogeneity. It includes multiphasic 3D implantable scaffolds and in vitro models, including systems that incorporate different regions emulating in vivo tissues, highlighting the emergence and relevance of 3D bioprinting in the future of biological research and medicine.

## Graphical abstract

When engineering tissues in vitro it is essential to mimic the heterogeneity present in native tissues, both in composition and biological components. While different approaches and manufacturing technologies are used in literature, bioprinting holds great promise, being able to recapitulate complex architectural 3D features and use bioinks to support biological requirements.

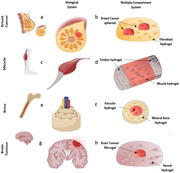

## 1 Introduction

Tissue engineering, a highly interdisciplinary field combining engineering, physics, chemistry, and biology, aims to create artificial constructs replicating the properties and behaviors of tissues and organs.^[^ ^1^, ^2^, ^3^ ^]^ Although a relatively young field, tissue engineering is moving at a fast pace, offering up solutions to some of the most pressing issues in current medicine and research.^[^ ^4^, ^5^ ^]^

At its inception, tissue engineering held the promise of revolutionizing the state of medicine by producing artificial tissues and organs on‐demand. Having a constant, infinite supply of organs offers an ideal solution to the shortage of organ donors, and patients requiring transplants could be treated with ease in a time efficient manner. This shortage and the need for a scientific solution to address patient demands^[^ ^6^ ^]^ is evident in recent data. According to a source from the USA, while the number of donors grew from 7000 to 19 000 in the last 30 years, the number waiting to receive an organ grew from 23 000 to over 112 000. This huge difference between demand and supply highlights the importance of increasing donor availability through the production of artificial organs.^[^ ^7^ ^]^ Despite successes with bone, cartilage, skin, and fat tissue transplantation,^[^ ^8^, ^9^ ^]^ organ transplants involving internal and sensory organs still require significant developments.^[^ ^10^, ^11^, ^12^ ^]^ This is mainly due to the complexity native tissues exhibit in their architecture, composition, heterogeneity in chemical and physical cues, intricate separation, and interaction of different cell populations. Even if artificially producing a construct capable of successfully achieving the aforementioned features was accomplished, the complete integration into a single fully functioning artificial tissue or organ would still prove a significant challenge.

With ongoing advancements in the field, it has become apparent that artificial tissues and organs will also assist in the production of artificial systems to provide controlled environments, offering a new platform to study biological systems both for fundamental and translational research, in addition to drug discovery and development.^[^ ^13^ ^]^ On average, an investment of around 800 million dollars and more than 13 years of research are needed between preclinical and clinical trials before a new drug reaches the market.^[^ ^14^ ^]^ There is, therefore, a strong demand for researchers to develop new approaches for effective drug screening and to improve understanding of related biological processes, like disease or organogenesis.^[^ ^15^, ^16^ ^]^ By introducing preclinical assessment tools halfway through the drug development process to identify potential efficacy and the risks associated with higher sensitivity, costly late‐stage failures could be avoided and wrong potential candidates could be identified and withdrawn.^[^ ^17^ ^]^

Preclinical assessments are currently performed using in vivo animal models, resulting in the use of an estimated 100 million vertebrate animals annually in research.^[^ ^18^, ^19^ ^]^ In addition to the obvious ethical concerns, these in vivo trials have proven to be less effective than their human in vitro counterparts, as they fail to appropriately reproduce human‐associated host responses.^[^ ^20^, ^21^ ^]^ In vitro tissue/organ models that could mimic and provide more accurate drug‐host predictions, while accelerating drug development and personalized medicine approaches, need to be created.^[^ ^22^ ^]^

As previously mentioned, artificial tissue/organ models are not only needed for drug discovery and development but allow for a more comprehensive understanding of biological processes resulting in the advancement of fundamental and applied research. For instance, artificial tissue/organ models could help better understand specific processes such as bone formation or remodeling; disease processes such as metastasis, diabetes, or neurodegeneration; or processes involved in organogenesis, like the formation of the villi in the intestine or the convolutions of the brain.

### 1.1 Biological Systems: Compartmentalization and Heterogeneity

In biological systems, multiple compartment complex architectures can be found in almost any organ present in the body. In fact, breakthrough technologies such as 3D bioprinting have shed some light onto our understanding of the highly complex material combinations, cells, and architectures that are present, and help support appropriate organ function.^[^ ^23^, ^24^, ^25^ ^]^ Even nonvascularized tissues, such as osteochondral tissues, show a gradient of mechanical properties from bone to cartilage, aside from different cellular organizations. In the brain, the cortex is composed of different layers with different compositions of neural types and extracellular matrix (ECM). In the musculoskeletal system, tendons, muscles, and bones have heterogenous architectures, which include rigid and elastic components.^[^ ^26^ ^]^ In kidneys, in addition to the stromal components, the renal pyramids include vessels from the circulatory and excretory systems.^[^ ^27^ ^]^ In the liver, lobules show a highly heterogenous architecture containing hepatocytes, Kupffer cells, veins, arteries, and biliary duct cells, as well as stromal components like fibroblast, all occupying specific regions of the functional unit.^[^ ^28^ ^]^ Bones, which are also vascularized tissues, possess a core region that contains the bone marrow, surrounded by a tougher region composed mainly of mineralized bone, with specific cell types in each of these regions. The bone marrow possesses different physicochemical properties to that of mineral bone and hosts a different population of cells, being essential in producing red blood cells that transport oxygen; white blood cells, to protect the body from infection; or platelets to prevent bleeding. Within the bone marrow, niches that exhibit different physicochemical properties, as well as different biological components can also be found. Similarly, osteons, which form the minimal functional unit of bone, have vascular channels surrounded by mineral components. The presence of these vascular channels is essential in providing appropriate nutrient availability throughout the bone.^[^ ^29^ ^]^ Likewise, the intestine shows highly heterogeneous architecture with separated compartments containing different cell types and contributing to different properties within the intestinal wall. For instance, the villi are formed by an epithelial layer that provides a barrier function, key to appropriate nutrient absorption, while underlying muscle layers contributes to the overall structural integrity and motility of the intestine. The lack of either of those components would result in a disruption of correct gastrointestinal function.

Given the intrinsic complexity in biological systems, it remains a challenge to engineer constructs that consider the heterogeneity, with patterned microarchitectures, that mimic native complex structural and functional units.^[^ ^30^ ^]^ In reference to the heterogeneity within complex biological systems, some of the most recent research aims to introduce different compartments or regions within a construct.^[^ ^31^, ^32^, ^33^ ^]^

### 1.2 Mimicking Biology In Vitro

**Figure 1:**
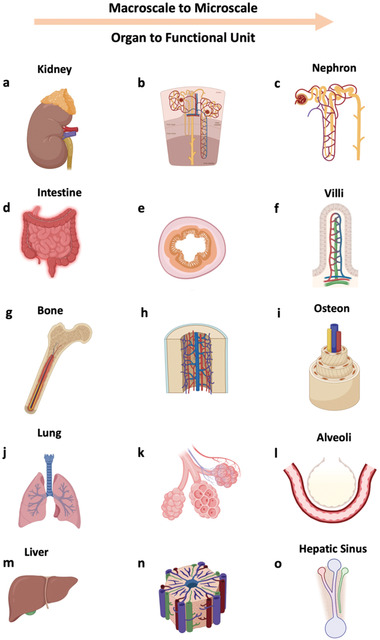
Multicompartmentalization and heterogeneity in biological systems. From top to bottom (different systems) and left to right (reducing scale): kidney to nephron, intestine to villi, bone to osteon, lung to alveoli, liver to hepatic sinus. Figure made with BioRender.

Traditionally, in vitro models started as specific cell types in 2D conformations, in an attempt to study organism behaviors outside their biological contexts. This evolved to research that used a combination of cell types on the same 2D surfaces, later progressing to more advanced 3D single cell type or multiple cell type cultures as a result of the increasing development of biomaterials, culture hardware and technologies, like micropatterning, microfluidics or 3D bioprinting.^[^ ^34^ ^]^ This in turn has brought the biomimicry of the designs one step closer to the desired physiological systems. By including ECM‐like material in in vitro models, the study of both cell–cell and cell–matrix interactions was made possible. Despite these developments, a suitable model to accurately replicate key features of human tissues is still not available.^[^ ^15^, ^35^, ^36^, ^37^, ^38^ ^]^ As a general rule, the closer a given model is able to replicate the targeted tissue architecture, the more accurate the predictions of function will be. Native tissues additionally have different regions with differing physicochemical, biological, and topological features, that are essential for the wider system and consequentially in the corresponding artificial tissue models created to mimic specific behaviors (**Figure** **1**).^[^ ^39^, ^40^ ^]^ As tissue function is inherently linked to tissue architecture, be it ducts, tubes or complex organizations, it is essential to replicate this native architecture to best achieve tissue specific functions.^[^ ^41^ ^]^ For instance, in liver, architectural organization is believed to be essential to hepatic function.^[^ ^4^, ^42^ ^]^

**Figure 2:**
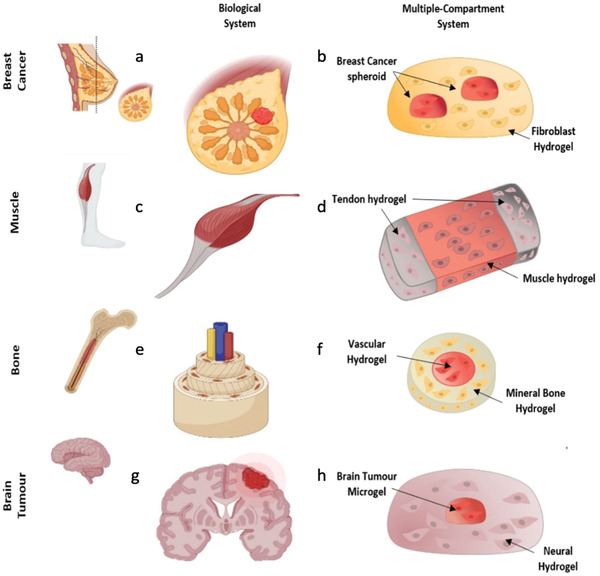
In vitro modeling, single and multiple compartment in biological systems. First row: a) breast cancer biological system component versus b) breast cancer cells‐based spheroid (parenchymal) within a fibroblast‐laden gel. (stromal). Second row: c) muscle biological system component versus d) muscle mimicking construct with tendon region at the sides to provide anchorage and a more rigid environment. e) Bone biological system component versus f) homogeneous mixture of stem cells and vascular cells. g) Brain tumor biological system component versus h) brain tumor model where brain tumor microgels (parenchymal) are embedded in neural hydrogel (stromal). Figure made with BioRender.

Multicompartment constructs, developed for both implantation and development of in vitro models, provide complex environments with multiple compositions and defined spatial control, by taking into account the inherent heterogeneity of native tissues (**Figure** **2**).^[^ ^43^, ^44^, ^45^ ^]^ Depending on the cell type and system requirements, the compartmentalization approach allows for independent customization of each compartment with regards to material composition, mechanical properties, and cell instructive properties.^[^ ^46^ ^]^ Furthermore, compartmentalizing allows for different regions of the construct to be subjected to different culture conditions and interactions. This is an alternative concept to multimaterial interfaces, which focuses more on creating systems where different materials are combined to create a hybrid environment with complementary or additional properties, e.g., thermoplastic microparticles to increase the stiffness of a gel or the combination of a first soft and ductile gel with a more rigid second gel that might be too brittle on its own.^[^ ^47^ ^]^

Compartmentalization also allows to create dynamic culture environments essential in emulating to a greater extent what exists in native tissues.^[^ ^48^ ^]^ Using this approach, researchers have been able to generate hypoxic conditions in the center of a model, forcing necrotic tissue formation with surrounding palisading cells to simulate the inside of tumors.^[^ ^49^ ^]^ Similarly, there have been examples where compartmentalization has been used to establish architectures in which parenchymal and stromal cellular components are able to interact, as seen in native breast cancer and liver.^[^ ^50^, ^51^ ^]^

Many of these models use a set of hydrogels as the base material to further increase tissue resemblance by providing a 3D ECM‐like environment. These hydrogels are often tailored using additional proteins and growth factors (GFs). Such cues, both physical and chemical, provide cells with an environment much closer to that of native tissues and are crucial in driving certain behaviors and phenotypes.^[^ ^52^ ^]^ As a rough classification, these hydrogels can be natural or synthetic and choosing which hydrogel type to use is an essential decision one needs to make after considering the relevant advantages and the disadvantages of each. For example, despite natural hydrogels usually being inherently biocompatible, they can carry some immunogenicity and interactions between cells and materials may be difficult to isolate. Although synthetic hydrogels can allow for isolation and fully characterized interactions between cells and materials, they have also been seen to carry higher toxicity due to toxic by‐products.^[^ ^53^, ^54^ ^]^ Additionally, dECM‐based hydrogels (obtained from the ECM of specific organs and tissues) can drive improved cellular responses,^[^ ^55^, ^56^, ^57^ ^]^ although the complex mix of proteins present can make them less suitable in systems where specific interactions need to be targeted or avoided.^[^ ^58^ ^]^

Most tissues and organs possess channel networks, be it for blood or air transport, to absorb nutrients, mediate secretion, or to provide immunological responses. Channel networks are therefore essential features to consider when aiming to replicate tissues and organs. When trying to produce constructs that mimic organs containing these networks, it is crucial that both the presence, location and shape of these vessels are considered.^[^ ^59^ ^]^ However, the fabrication of small hollow tubes remains a challenge that is only recently being tackled through the development of new 3D bioprinting modalities, like stereolithography. These vascular network compartments are typically placed within large, solid compartments that represent the surrounding environment within that tissue or organ. Often multiple networks will be used in the same construct to emulate more complex neighboring channel structures within a tissue, as seen in the pancreas or the kidney. For instance, recent work used a double network embedded into a hydrogel to imitate a blood vessel and renal tubule, which was used to study the renal absorption processes. Similarly, researchers engineered a pancreatic ductal cancer model that recapitulated the crosstalk between both a cancer duct and a neighboring blood vessel. In addition to increasing the structural similarity with native structures, these networks provide essential nutrient, protein, or drug distribution within these bigger compartments, and are thus able to replicate essential functions, such as renal tubule absorption or gas exchange in the lungs.^[^ ^60^, ^61^, ^62^ ^]^

To sum up, when engineering biomimetic constructs there are some challenges that need to be tackled to replicate their native environment. For instance, cell density will vary between tissues: adipose tissue is mostly ECM, while endothelia are formed by densely packed cell layers essential to provide barrier function. Moreover, appropriately replicating native ECM properties and composition is a topic of great interest. As a result, materials like hydrogels, that are made of decellularized native ECM, or that incorporate proteins like fibronectin or laminin, or other biomolecules like GFs have been developed. Not only that, but some biological systems can also be modeled in microscale while other exist in bigger scales (several cm). On that same line, some tissues or organs might possess complex morphologies and architectural features like cavities or channels. Similarly, some tissues can be found under more “static” conditions, like the skin; while others experience flow or mechanical stimuli, like blood vessels or bones and the lungs, respectively.

The closest these native conditions can be replicated, the closer the response of the engineered construct should be to that of the native system. However, it is important to note that sometimes, culture conditions and systems can be simplified as much as desired, to have more control and isolation over the interactions happening within the system. This is the case of some two‐dimensional (2D) models, which allow to engineer and understand essential processes within tissues and organs without all the complexity that other more complex 3D models might have.

This paper reviews some of the latest developments in multicompartment constructs for tissue engineering. It discusses not only the types of design and architecture approaches that have been developed in recent years to create multiphasic, compartmentalized models but also the varying fabrication techniques and technologies that have been adopted. Amongst these, 3D bioprinting emerges as a key technology that has the potential to dramatically shift the current state of medicine, pharma, and biological research.^[^ ^63^ ^]^

## 2 Approaches for Multicompartment Models

**Figure 3:**
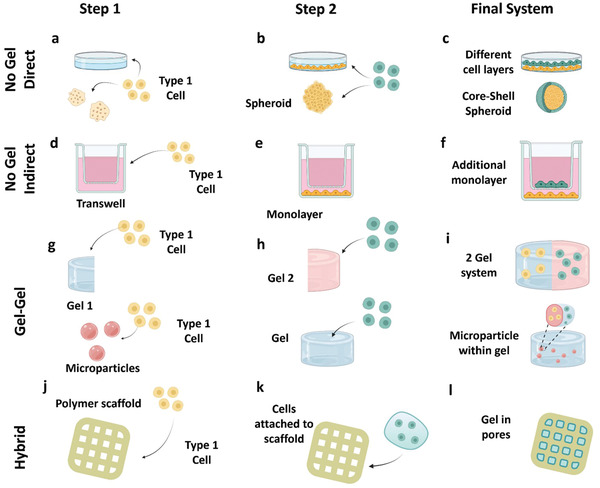
Approaches for compartmentalization. First row: NoECM direct contact. A first compartment is created, once the initial conformation is achieved a second compartment might be formed over it. Second row: NoECM indirect contact. A first compartment is created, within the same culture space a second compartment is created allowing them to secrete signaling molecules but without direct contact between them. Third row: gel–gel. A combination of different hydrogels leads to a two or more hydrogel system. Hydrogel microparticles can be used to obtain compartments in different scales. Fourth row: hybrid multimaterial approach, where a combination of thermoplastics, cements or hydrogels might be used together in order to generate the different compartments. Figure made with BioRender.

Multicompartment models and constructs aim to replicate, to the highest degree possible, native tissue heterogeneity. This replicability is an essential criterion for artificial constructs to display behaviors inherent to specific biological systems. A wide range of designs and conformation type have been developed to engineer constructs that possess separated compartments, mimicking specific regions within native tissues. These approaches, however, differ from each other in relation to the materials, technologies or workflows used to achieve these compartments in the final construct (**Figure** **3**).

### 2.1 No Gel Direct Contact

The first type of conformation does hold limited structural requirements to achieve compartmentalization, but still allows for a series of compartments to be created throughout the construct. This type of conformation will be referred to throughout this review as direct contact. Direct contact relies on the formation of compartments via cells forming an initial monolayer, multilayer or spheroid, which is allowed to fully form and establish before a second layer of cells is then added. The first compartment can be formed over a biomaterial that facilitates the arrangement of cells in the desired configuration. A second cell type is then added to the construct, forming a new compartment, creating a second layer over the first (Figure 3).^[^ ^64^ ^]^ In this case, the creation of compartments relies either upon the attachment capabilities of cells or the ability to place a group of cells that have already formed some degree of structure inside or adjacent to an additional material.^[^ ^65^, ^66^ ^]^ It is important to note that both cell layers are in contact, allowing for a very close communication via both paracrine and juxtracrine signaling.

For instance, a nucleus pulpous‐cartilage model was created by timing the addition of various cell types over a calcium phosphate structure, i.e., to enable cartilage formation, a porous calcium phosphate support structure was seeded with chondrocytes prior to adding nucleus pulposus cells to complete the construct. Adding the cartilage phase to the system was found to improve tissue attachment and mechanical performance of the construct, although the system could further benefit from using more physiologically relevant materials like hydrogels and ECM components such as collagen or hyaluronan.^[^ ^67^ ^]^ Other studies have fabricated human perivascular bone models to examine metastatic colonization. In these models, a first bone component filled with bone marrow‐derived mesenchymal stem cells (MSCs) and endothelial cells was created prior to adding breast cancer cells one week later to obtain a triculture system. The model was able to reproduce stable vascular networks and showed similar effects of the drug sunitinib to those in vivo, i.e., a lack of effect over slow‐proliferative breast cancer cells, which are suspected to have a role in metastasis and colonization. Metastasis might, however, be mimicked to a higher degree by separation of the cancerous compartment and allowing cancer cells to migrate toward the perivascular compartment instead of a direct injection of cancer cells into the perivascular model.^[^ ^68^ ^]^

As stated above, functional variations were achieved by placing a previously arranged structure inside a defined material to obtain the final multicompartment construct. One such example is the formation of pancreatic cancer organoids, where premade pancreatic cancer organoids were introduced into a hydrogel system that contained fibroblasts and/or lymphocytes. This design displayed increased resistance to treatments compared to organoid‐only systems.^[^ ^69^ ^]^ It is also worth mentioning that, in some cases, depending on the attachment strengths and self‐assembling abilities of cells, heterogenous populations of cells have been able to form compartments without the need of an external arrangement. For example, core–shell pancreatic cancer spheroids were obtained by seeding pancreatic cancer cell lines and pancreatic stellate cells (PSCs) at various ratios. Here, the spheroid replicated PSC microenvironment within pancreatic ductal adenocarcinoma and displayed chemoresistant, invasive, and metastatic phenotypes.^[^ ^70^ ^]^

3D liver models were also created with this approach through seeding human umbilical vein endothelial cells (HUVECs) on top of a collagen‐coated paper. After the initial monolayer formed, a second component of induced pluripotency hepatocytes (HiHeps) were added. This set up maintained a higher expression of certain liver‐associated genes compared to monolayer constructs, in addition to the support of liver specific functions such as albumin secretion or urea synthesis for two months. Despite this, hepatic function appeared to reduce over the two‐month period of the study. A more physiologically relevant environment or greater compartmentalization could help mitigate the loss of hepatic function.^[^ ^71^ ^]^

The main advantage of this direct cell contact technique is its simplicity; it does not necessarily rely on complex materials or additional fabrication processes. In fact, it only requires a first cell type to be cultured in the appropriate format, be it a monolayer, spheroid or within a polymer scaffold. Once the required time has passed and the desired structure has been achieved, a second cell type can be added or the structure can be transferred onto a different system, such as a 3D hydrogel. This greatly reduces the level of difficulty and precision required in creating compartments, providing a more approachable take on multicompartment constructs. This is also supported by the process being less technically demanding as there is no need for extra equipment such as microfluidics, 3D bioprinters or patterning systems in its creation. By having cell‐only compartments in such proximity with other cell‐only compartments, this type of model also facilitates interaction and signaling between them. However, while some of the generated constructs might be considered 3D, this approach often disregards the contribution or importance of ECM‐like material embedded into the final construct, ignoring key design principles such as appropriate tailoring of composition, mechanical cues, or chemical cues to that of corresponding native tissues.

### 2.2 No Gel Indirect Contact

The second approach, which will be referred to as indirect contact, follows a very similar principle to the first approach. However, instead of allowing different cell compartments to form on top of or surrounding each other, cells are allowed to form next to each other at opposing sides of a membrane, which can simultaneously have differentiated culture spaces (specific media and conditions) or not. In these models, cells are still in 2D, reducing the experimental difficulty while still allowing for the controlled study of biological interactions and the importance of differing components of a certain system.

When cells are placed immediately next to each other, there inevitably will be some direct contact between cells of adjacent compartments, however, most of these cells will only benefit from secreted factors by cells in either compartment. For instance, Khetani and co‐workers engineered an in vitro model that improved the maturation and longevity of hepatocytes by culturing them in compartments surrounded by fibroblast containing compartments. However, the presence of undesired markers was still present and further integration of chemical cues would improve the effectiveness of the model.^[^ ^72^ ^]^

In other set ups, cells are placed in the same shared culture environment in two different compartments, created by separating the two cell populations via a membrane, i.e., a transwell model. Transwell models put emphasis on separating cell populations to allow them to form and often develop into a barrier‐like monolayer. Lau et al. developed a transwell model that allowed the study of intravasation of breast cancer cells and model entrance into circulation by isolating breast cancer cells on one side of a Matrigel layer that had an endothelial monolayer on the opposing side. This model allowed for the identification of specific breast cancer populations that were then consequently used in in vivo models. This study provides a good example of how multicompartment in vitro models could help to improve drug development and facilitate fundamental research by offering a first evaluation of potential candidates before moving to animal models.^[^ ^73^ ^]^ Another model, used to study cardiac tissue, consisted of cardiomyocytes and fibroblasts at either side of a poly(lactic‐co‐glycolic acid) PLGA membrane. This cardiac model facilitated the interaction between fibroblasts and cardiomyocytes, and showed enhanced cardiac reprogramming of the fibroblasts at both gene and protein level when electrical stimulation was present. While this is an interesting development, due to its small scale and low‐throughput, this system might be better suited to study the molecular mechanisms by which cardiac‐reprogramming happens rather than to provide a cardiac cell source as originally intended.^[^ ^74^ ^]^

The drawbacks of transwell models include the inability to investigate the effect of shear flow or dynamic culture conditions in addition to having different culture conditions for each compartment. This can be solved by including the different cell populations in different chambers of microfluidic devices (which will be explained in depth within the next section of this review), separated by permeable or discontinuous barriers. Using this technique, researchers have engineered microfluidic platforms that allow for the study of interactions between osteoclasts and osteocytes and the implications of flow in those interactions. The researchers were able to show increased osteoclast differentiation toward nonstimulated osteocyte channels due to the increase of secreted factors such as Receptor Activator for Nuclear Factor k Ligand (RANKL). However, the system has a very low‐throughput and does not include oscillatory fluid flow which would enhance its relevance as bone suffers cyclic loads as opposed to constant loads.^[^ ^75^ ^]^ Rahman et al. used a similar platform (although under flow‐free conditions) to evaluate intercellular communication between breast cancer cells and adipose cells. The platform showed not only enhanced growth and proliferation, but also resistance to gold‐standard drug treatments when breast cancer cells were cultured with adipose cells. This suggests that factors secreted by adipose cells play a key role in breast cancer growth and drug resistance. Despite these positive findings, the inclusion of mechanical or chemical cues, and embedding the cells in relevant 3D culture platforms could greatly improve the biomimicry ability of the model.^[^ ^76^ ^]^ Other models involving this type of set up were used to study the neuromuscular junction. Neurons and muscle cells were placed at either side of a barrier (consisting of microgrooves), allowing for communication and the invasion of neurons into the muscular compartment. The ability to manipulate each compartment in fluidic isolation individually allowed for a closer specific environmental match. This has important implications as it facilitates the study of the effect of targeted treatments and may contribute in developing a better understanding of neuromuscular processes and neurodegenerative diseases.^[^ ^77^, ^78^ ^]^

While this second approach is similar to the first, here, the majority of the contribution of the effect of one cell on another is via secreted factors that allow for indirect communication rather than direct communication as seen previously. Given the nature of 2D culture systems, the level of complexity of this approach is lower than that of its 3D counterparts. 2D systems do not take into account the embedded nature of the cells within a complex environment that is able to guide and instruct cells into specific phenotypes and behaviors. However, they are still able to mimic essential separations and compartmentalization of native tissues, and present enhanced responses and advantages over traditional monocompartment monocultures or cocultures.

### 2.3 Gel–Gel

The next approach uses hydrogels: highly hydrated polymer networks. These materials have ≈90% water content and provide a highly suitable 3D environment for cells to live and thrive in, by closely mimicking conditions in which cells can be found in organs. Most importantly, these hydrogels can either be made up of, or functionalized, with ECM properties and proteins to enhance the resemblance of the matrix to that of the physiological organ.^[^ ^79^ ^]^

In this approach, the constructs in which compartmentalization was achieved by combining two or more hydrogels in spatially distributed configurations have been included. Traditionally, this was done using casting and molding techniques, in which several of these compartments were assembled using complementary geometries or additional adhesives to keep them together. However, in recent years, 3D bioprinting has enabled fabrication of each of these compartments simultaneously, consequently introducing the creation of architectures with greater complexity.

Hydrogels are often combined to produce multiple compartments in either the horizontal or the vertical axis (Figure 3). Some models consist of an interior hydrogel that is then fully surrounded by a second material in an enclosed sphere configuration. For example, in one study, a pancreatic islet containing hydrogel was fully surrounded by a vasculogenic hydrogel to obtain implantable devices with mechanical and proteolytic stability and an increased support of islet viability. The encapsulation inside the vasculogenic compartment improved islet viability when compared to control single‐compartment gels, however, vascular endothelial growth factor (VEGF) incorporation was also seen to be instrumental in the observed effect.^[^ ^80^ ^]^

This approach, despite having higher requirements in terms of structural stability as well as fabrication complexity when compared to the above described, can more closely resemble the native architectures found in tissues and organs than other approaches. In addition, the presence of hydrogel components provides the ECM‐like environment that grants cells with a closer microenvironment to that of physiological systems. Osteon‐like environments were created by combining MSCs and HUVEC cell‐laden fibrin gels using a concentric ring design which contained the cells in outer and inner rings respectively. This resulted in constructs that recreated an optimal microenvironment by mimicking the native cell pattern found in bones, specifically mineralized bone surrounding the Haversian channels. However, while in in vitro this showed increased angiogenic potential, little work was done in vivo. This system, which was initially intended for implantation, could prove a useful tool in studying essential bone‐related processes and diseases.^[^ ^81^ ^]^

Furthermore, this approach allows for a subsequent control of the culture conditions by combining the constructs with microfluidics or flow‐containing bioreactors, as well controlling parameters such as hypoxia or chemical gradients. This enables the fabrication of highly complex models that not only support compartmentalization but also enable compartments to work under different conditions, further increasing progress toward the biomimicry of native tissues.^[^ ^82^ ^]^

By horizontally patterning several concentric rings, including both tumor and endothelial components inside a silicon chip, high fidelity glioblastoma (GBM) models have been created. This glioblastoma on a chip included the GBM component surrounded by a blood‐vessel containing layer often found in tumors. In this silicon chip, additional conditions such as hypoxia were generated in the center of the model, thus incorporating native glioblastoma features such as the existence of a necrotic center surrounded by pseudopalisading cells. When patient‐specific models were generated, the results resembled the observations made in clinics, thus offering hope for these models to be used in assessing the potential effect of different drug candidates and treatment combinations.^[^ ^49^ ^]^

Vertical patterning of different properties of hydrogels containing the same cell type were used to mimic osteochondral tissue, where a bottom hydrogel layer with high stiffness values was topped with two layers of decreasing stiffnesses, simulating the bone‐like component gradually turning into cartilage. This illustrates how the presence of multiple compartments and compositions, even if only physicochemical and not biological, provides an improvement in tissue resemblance.^[^ ^83^ ^]^

Importantly, gels are not required to be on the same scale within a model. Cell‐laden hydrogel microparticles embedded within a carrier hydrogel are an example of this. In this case, the incorporation of these microparticles results in multiple compartments at a small scale. Be it different polymers, biomolecules, GFs or cell types, these microparticles together provide a different environment to that of the bulk hydrogel (Figure 3). Additionally, several microparticles with differing compositions can be spatially arranged in different areas of a construct, offering varying compartmentalization features. Microparticle fabrication processes have previously been engineered to produce enough spatial distribution to generate separate areas containing different cell types^[^ ^84^ ^]^ or chemical profiles.^[^ ^85^, ^86^, ^87^ ^]^

This approach has been used to allow for cross‐scale control over tissue‐mimetic features in the design of culture systems that consider mesoenvironmental characteristics, i.e., both a macro‐ and microenvironment. A novel prostate cancer model including cancer spheroid microgels, surrounded by a network of HUVEC capillary structures, has been created utilizing this. The approach showed great potential, providing vascularized multicompartment embedded prostate cancer spheroids. Despite this, as the authors suggest, the paper describes a proof‐of‐concept approach and specific control of features such as microgel size (to control interfacial area between compartments) or mechanical properties of each compartment should be implemented to better understand processes such as tumor progression. In the future, this system could be used in other coculture and organoid schemes that could also include several types of these microparticles positioned in different areas of the construct.^[^ ^88^ ^]^ Some implants use microparticles inside a bulk hydrogel. This provides an environment for very sensitive cells in which phenotypes could be maintained for longer in comparison to bulk cell‐laden hydrogels. For instance, a study where a combination of support microparticles (aimed at improving graft acceptance) and pancreatic islets inside an injectable hydrogel were injected in diabetic mice, found close to a threefold increase in graft acceptance over 200 days while maintaining function by normalizing glucose levels. This result cannot be attributed to the presence of a multicompartment alone, but instead as an addition to the appropriate functionalization of the microparticles that provided an essential immunomodulatory effect when islets‐containing hydrogel grafts were implanted.^[^ ^89^ ^]^

Previous works following a very similar approach have achieved a better reproduced drug response to patient‐specific treatments by using a spheroid inside a cell‐containing gel strategy, including both pancreatic cancer and stromal cells within confined areas.^[^ ^90^ ^]^ A particularly interesting feature of this approach is the possibility of producing microparticles that can be made entirely of different hydrogel systems, without the need to incorporate similar materials or glues in order to bind different compartments, which are typically otherwise needed. Microparticles additionally offer conditions that create multiple tiny compartments that could potentially support nodules and host smaller populations of cells in similar ways to some native tissues that possess dispersed and localized populations, i.e., pancreatic islet in the pancreas.

### 2.4 Hybrid

Similar to the previous approach in which two or more hydrogels are combined, two or more materials can be combined to obtain multicompartment constructs. In this second approach, however, it is not a combination of two or more hydrogels but rather a combination of hydrogels with thermoplastics or cements (Figure 3).

Even though hydrogels provide a friendlier environment for cells in terms of toxicity and ability to replicate a highly hydrated microenvironment, the mechanical properties are much lower than those of thermoplastics^[^ ^91^ ^]^ or cements.^[^ ^92^, ^93^ ^]^ In fact, stiffness values of hydrogels, which researchers have been able to control and tune to best fit their applications, vary from below 100 Pa to over 100 kPa.^[^ ^94^, ^95^, ^96^ ^]^ While these are the ideal mechanical requirements for tissues like vasculature, brain, or soft organs, it is often insufficient to mimic the properties of structural tissues. Trabecular bone, for example, has an elastic modulus varying from 6 to over 20 GPa in humans depending on the specific bone under study and the scale used with the applied measuring technique.^[^ ^97^ ^]^ As a result, this approach has been mostly used in tissues that can be found in the musculoskeletal system such as bone, cartilage, muscle or tendons.

To provide biomimetic mechanical properties while keeping a cell‐friendly environment, a urethra model was printed consisting of a multiphasic tube, combining an inner region of urothelial cells embedded in a hydrogel, with a middle region made of polycaprolactone/poly(lactide‐*co*‐caprolactone) (PCL/PLCL) and an outer region of smooth muscle cells embedded in a hydrogel. The resulting bioprinted construct exhibited appropriate viability and proliferation of both cell types and had mechanical properties like that of native urethra. The study, however, only took place over one week of culture and hence failed to elucidate biological responses at longer time points. Despite this, the study demonstrates how in vitro analyzing and modeling may serve as an essential step in the evaluation of artificially engineered constructs, which could further be translated to in vivo studies and ultimately used as implants for both animals and humans.^[^ ^98^ ^]^

To obtain the same integrity of native tissues, it is crucial to provide the structural elements of the mineral component of bone, and the enthesis or anchor points in muscles. A muscle–tendon unit was engineered by combining a polyurethane region, a coprinted hydrogel with C2C12 cells, acting as the muscle, with a polycaprolactone (PCL) region, a coprinted hydrogel with NIH3T3 cells, acting as the tendon. This allowed for the fabrication of a scaffold with both elastic and stiff regions, while maintaining good viability, highly aligned morphology, and increased muscle–tendon‐associated gene expression. Further addition of biomechanical cues, such as stretch–relaxation, could greatly influence the impact of this model, allowing for a better understanding of regeneration and biological processes involving muscle and tendon structures by studying them in a more biomimetic context.^[^ ^99^ ^]^ Similarly, an osteochondral region was engineered by combining an MSC‐seeded PCL region, with an MSC‐laden gelatin methacryloyl (GelMA) hydrogel region. This construct was then subjected to osteogenic media for the PCL region, and chondrogenic media for the GelMA region. The PCL region was later coated with an HUVEC‐laden GelMA hydrogel and allowed to vascularize. The resulting osteochondral construct was able to mimic both structural architecture and gene expression profiles.^[^ ^100^ ^]^

Bone models have utilized this approach by incorporating a structural material to act as the mineral component and a second hydrogel‐based material to provide the environment for the cells. A multicompartment osteogenic model included a PCL scaffold with a series of interconnected pores filled with different variations of cell‐laden gellan gum hydrogels. MSC hydrogels were placed in the inside and outside pores, while the interconnecting pores were seeded with endothelial cell hydrogels. Due to this compartmentalization, the osteogenic effect and cell viability were seen to improve compared to the mixed and monoculture controls. However, the structure and cell distribution of the model could be redesigned in future to feature a more native bone‐like architecture and enhance the biomimicry of the model.^[^ ^101^ ^]^ In a similar work, combinations of PCL and decellularized ECM‐based bioinks were coupled to allow the PCL to enclose each line of printed bioink. Using this method cartilage, cardiac and adipose tissue analogues were created, displaying high viability and functionality.^[^ ^102^ ^]^

In a humanized osteosarcoma model, a tube of porous melt electrowriting PCL scaffold coated with calcium phosphate (CaP) microparticles was seeded with MSCs before being osteogenically stimulated over a period of seven weeks. It was combined with HUVEC‐laden PEG–VEGF hydrogel seven days before implantation to the murine host to allow pre‐vascularization of the construct and generation of a tissue engineered bone construct (TEBC). Another inner compartment composed of recombinant human bone morphogenic protein‐7 (rh‐BMP7) fibrin gel and laden with CD34+ cells, was used as the “glue” between the murine bone and the TEBC to establish a humanized hematopoietic and immune system. As a result, they were able to create a model, which included both bone matrix and bone marrow, meeting of all the requirements for use as a patient‐specific in vivo testing platform.^[^ ^103^ ^]^

## 3 Manufacturing Technologies

**Figure 4:**
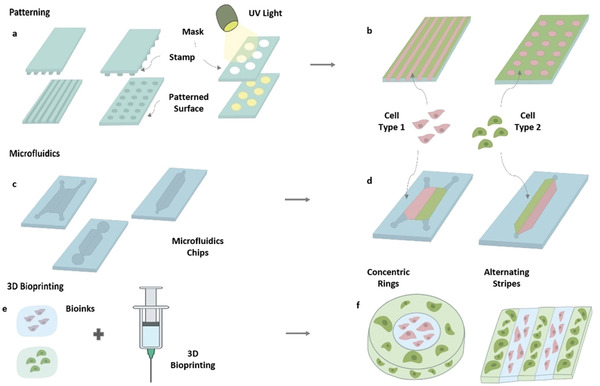
Technologies used for fabrication of compartmentalized models. a) Masks and Stamps are used to create patterned surfaces with indented or differently treated compartments. b) Different cell types are placed on different compartments to create multiple juxtaposed compartments. c) Microfluidic chips can be produced with neighboring compartments. d) Different cell types with or without support materials can be placed in each of these compartments. e) Bioprinting is able to combine several bioinks within the same construct, creating complex architectures that include f) different materials and cell types.

Aside from analyzing different approaches to achieve multicompartment constructs, it is also important to pay close attention to various manufacturing technologies employed to obtain these constructs (**Figure** **4**). Advances in these types of constructs have been strongly driven by advances in adjacent biofabrication technologies including micropatterning, microfluidics, and 3D bioprinting. Despite the differences amongst these techniques in terms of the technology adopted, all of them are capable of providing constructs with clearly differentiated compartments throughout the structure. However, the type of materials they use, the final configuration of the construct, 2D or 3D application, and the precision and scale that can be obtained vary greatly between them. The choice of the technology ultimately depends on the type of application, the biological system, and the level of complexity required. For instance, 2D models typically have a lower complexity than 3D models, some organs are more vascularized than others, and some biological systems might require the presence of channels to properly mimic the system under study.

### 3.1 Micropatterning

Micropatterning is the oldest of many technologies used in the manufacturing of multicompartment constructs. Micropatterning allows for the creation of microscale patterns carrying varying properties in different regions, thus enabling different cells to be added to specific regions of the surface. Although 2D configurations are common, some examples of micropatterned 3D structures can also be found. These are typically made by stacking several 2D micropatterned structures in the vertical axis, or by using hydrogels.^[^ ^104^ ^]^

The most well‐known micropatterning technology is soft lithography, which refers to a collection of techniques that enable pattern fabrication using stamps, molds, and photomasks. Stamps enable the creation of slits and indentations in structures, while molds allow for the creation of a complementary structure (positive) out of the chosen material. Photomasks selectively permit light through, enabling the production of structures using photocurable or photoinducible materials (Figure 4).^[^ ^105^ ^]^

PDMS stencils, PDMS stamps or selective light blocking masks have been widely used to obtain some of these patterns. In a microscale model of human liver, an initial PDMS stencil was used to generate equidistant circles that were then treated with collagen before seeding with hepatocytes. After removal of the PDMS stencil, fibroblasts, chosen to promote induction of liver‐specific functions in hepatocytes, were added to populate the remaining surface, acting as the stromal component of the stromal‐parenchymal tandem. The tandem led to a higher level of hepatic functions compared to conventional gel matrixes.^[^ ^106^, ^107^ ^]^ Similarly, coculturing parenchymal cells surrounded by fibroblasts expressed liver‐like molecules inducing human hepatocyte function.^[^ ^108^ ^]^ Long‐term stability of hepatic functions was also established using coculture models including liver and nonliver derived parenchymal cells.^[^ ^109^ ^]^ Another model for drug development using a triculture of hepatic stellate cells along with a fibroblast/hepatocyte layer,^[^ ^110^ ^]^ maintained a long‐term hepatic phenotype by coculturing hepatocytes and endothelial cells.^[^ ^34^, ^108^ ^]^ These liver models displayed increased hepatic function and could provide a better evaluation tool for the prediction of clinical outcomes compared to monolayer single compartment cultures. However, while they can somewhat mimic liver heterogeneity, these models still fail to recapitulate exact liver architecture and major advances are still required to fabricate models that will more accurately predict and replicate liver‐associated diseases and processes.

These patterns can also be treated chemically to allow certain types of cells to attach to the desired regions while avoiding cell adherence to other areas. Additionally, using consecutive masks enables several patterns to be created.^[^ ^111^ ^]^ Using this technique, a model with a mixture of adherent and nonadherent cells was created by selective treatment of different areas of the surface. Treating an area with albumin‐cell membrane anchoring reagent (CMAR) allowed typically nonadherent (NAd) cells to adhere. This allowed for nonadherent cells to couple with adherent (Ad) cells, thus enabling the study of the compartmentalized cocultures. This system was used to study a neuroblastoma (Ad)–myeloid (NAd) model, and a colon cancer (Ad)–monocyte (NAd) model to evaluate the effect of monocytes on drug sensitivity of cancer cells. Monocytes were seen to induce drug sensitivity of cancer cells against deoxy fluoroudine (DFUR). However, this effect was mostly limited to the cancer cells found within 1 mm of the monocyte source, highlighting the importance of the architecture over the displayed response of a model or any in vitro engineered tissue. The model could benefit from a different design instead of the proposed rectangular areas placed next to each other. Multiple circular regions placed inside a bigger square region would maximize the area of effect of monocytes over cancer cells, while at the same allowing the observation of whether multiple monocyte sources have an impact on the overall cancer cell response.^[^ ^112^ ^]^ In a similar fashion, another study used micropatterned collectors to obtain electrospun fibrous mats that were then organized together to create multicompartment models with highly intricate designs, to consider the interactions between three different cellular components in a cardiac model. Following the native cardiac tissue composition, cardiomyocytes were coupled with cardiac fibroblasts (to secrete ECM components, soluble factors, and transduce mechanical signals), and endothelial cells were included to promote vessel formation and secretion of endothelial growth factor. The researchers proved the importance of architecture design; particular architectures, such as a wave‐patterned configuration, displayed significantly higher cardiomyocyte elongation, alignment and beating rates when compared to other multicompartment constructs.^[^ ^111^ ^]^ Similar striped micropatterns were used to create hepatocyte‐fibroblast (parenchymal–stromal) liver models that allowed retention of hepatocyte phenotype and function.^[^ ^113^ ^]^

As previously described, micropatterning can also be done in 3D using hydrogels. Digital light processing (DLP), for instance, uses the same approach as lithography in which a photomask is used to generate a pattern, although here the photomask is used to selectively cure specific regions of bulk hydrogels.^[^ ^114^ ^]^ Other models used a polydimethylsiloxane (PDMS) stamp while curing upon a first compartment already containing cells to generate indentations, i.e., on a cell‐laden photocurable hydrogel. Once cured, a second cell type was deposited on the indentations created.^[^ ^115^ ^]^ However, presence of light was found to not be an essential requirement to create these patterned structures. Micromolding allowed the use of thermally or chemically crosslinkable patterns, thus avoiding the toxicity that comes with some of the light‐mediated techniques.

For instance, a highly vascularized, heterocellular 3D architecture, emulating a liver lobule was created as an in vitro model. The model had a center circular region, containing endothelial cells and a surrounding region containing hepatic cells. A PDMS mold was used and a patterned structure to which endothelial cells could be attached was created. Stacking these circular and stamp‐patterned structures was carried out to produce the final liver‐lobule, which consisted of both vertical and horizontal vascularization. The multicompartment architecture resulted in an increased urea production from hepatocytes, a useful measure of its effective function, compared to both hepatocytes in hydrogel gel matrices and un‐patterned cocultures.^[^ ^104^ ^]^

Another study used PLGA– poly‐L‐lactic acid (PLLA) to create macroporous structures that were then cut into different sized circles and seeded with a fibrin gel laden with fibroblasts and endothelial cells. A smaller circle containing endothelial cells was next placed inside the bigger fibroblast containing circle. This system was introduced in a multi‐flow channel bioreactor to induce endothelial barrier formation. Fibroblasts migrated to stabilize the EC region and thereby improved the formation of the vascular structure.^[^ ^82^ ^]^

In conclusion, micropatterning has been used to study a wide range of different systems, showcasing the potential of the technology. However, despite more recent 3D models obtained via DLP, the technique usually entails 2D structures in which the 3D component of tissues is overlooked. Referring to the previous section, micropatterning would be more suitable for an approach that includes non‐ECM indirect cell contact, although sometimes, ECM features are present as a surface treatment rather than by providing a 3D ECM‐like environment. As micropatterned models are typically done in 2D, there is a limitation in the achievable biomimicry capacity of the model. In order to appropriately mimic tissue heterogeneity, it would be more desirable to include this type of 3D configuration. Despite this, some of these models have provided valuable knowledge and an increased understanding of biological processes. Furthermore, the simplification into a 2D configuration has enabled the limitation of the number of variables involved when trying to elucidate biological processes or disease mechanisms.

### 3.2 Microfluidics

Microfluidics involves the use of chips made from glass or polymers (such as poly(methyl methacrylate) (PMMA) or PDMS) with microsize channels created to allow different solutions to flow through. This technology has gained great interest within life and chemical sciences due to the miniaturization these devices allow for. This key feature consequentially enables the study of phenomena that has previously escaped macroscale devices. The popularity of microfluidics is accelerating in tissue engineering with the birth of organ‐on‐a‐chip approaches. An example of this microfluidics system placed living cells along with other culture materials in different compartments to simulate tissues and organs, avoiding oversimplified traditional 2D models (Figure 4).^[^ ^116^ ^]^ Currently, there are models using microfluidics that emulate almost any organ present in the body and are used for both research and industrial applications.

Apart from providing this compartmentalization, microfluidic devices have an especially interesting property; the ability to generate and control a nutrient and biomolecule gradient throughout the model, thus allowing for a higher level of precision. This is done by incorporating perfusable channels that can distribute the desired solution using fluid pumps.^[^ ^38^ ^]^ Usually, cells are found in a material‐free context, often in 2D set ups, spheroids, or organoids, although some models include other material components such as hydrogels.

In lab‐on‐a‐chip and organ‐on‐a‐chip approaches, microfluidic chips that mimic a specific biological system allow for an efficient evaluation of fundamental biology, disease mechanisms or chemical compounds. This is due to the ability to replicate functional units of several organs, both structurally and chemically.^[^ ^117^ ^]^ For instance, a model was designed to study the degeneration of the corticostriatal circuit, a key neuropathological and clinical feature of Huntington's disease. Mimicking the corticostriatal circuit, by coupling a cortical and striatal neuron compartment, allowed identification of key proteins involved in both degeneration and regeneration. In this model, multicompartmentalization was used to achieve multiple key elements other than just the containment of the culture‐isolated population of the different neuron types. The 500 µm microgrooved spaces between compartments, through which standard synapses of both neuron types were displayed, enabled the development of a healthy corticostriatal circuit model. The modifiable nature of the model allowed it to emulate reduced synaptic connectivity and striatal neuron atrophy commonly associated with Huntington's disease. Finally, the identification of key chemicals involved in the process (brain‐derived neurotrophic factor) and the evaluation of the potential treatments to regenerate the diseased circuit were also made possible due to the compartmental feature of the model.^[^ ^118^ ^]^

Multicompartment tumor models have also been created using microfluidics, where tumor, stromal, and vascular cells were spatially organized and used to study morphology, growth, and the microenvironment.^[^ ^119^ ^]^ Another effort was made to create an early‐stage breast cancer in vitro model, using a coculture of breast tumor spheroids with human mammary ductal epithelial cells and mammary fibroblasts. This method aimed to replicate the microarchitecture of breast ductal carcinoma using a microfluidic chip made of two cell culture chambers separated by a membrane mimicking the native basement membrane. The upper chamber contained the tumor component, while the lower chamber contained the stromal component. While the system showed an appropriate response against gold‐standard drug treatments, it is still a simplified representation and fails to include other important cell types or controllable mechanical properties that may increase the resemblance to an in vivo context.^[^ ^120^ ^]^

Generally, different compartments inside a chip are connected or allowed to communicate by incorporating pores or permeable membranes as walls between them. The central feature of the work by Shirure et al. included endothelial cells placed between two cancer adjacent compartments.^[^ ^121^ ^]^ Similarly, a colorectal tumor‐on‐a‐chip model was created with an inner compartment containing colorectal cancer cells connected to an outer compartment containing endothelial cells via small channels, allowing the study of endothelial invasion into the inner compartment amongst other things.^[^ ^122^ ^]^ A liver‐on‐a‐chip system used a parenchymal compartment containing primary hepatocytes interfaced with a vascular channel containing endothelial cells with or without Kupffer and hepatic stellate cells. The model was later used to detect different liver toxicity phenotypes. In this work, they develop three species‐specific liver‐on‐a‐chip, all of which showed different hepatic function and marker differences. Compared to other models, such as the micropatterned liver models described in the previous section, these liver‐on‐a‐chip models were able to mimic the dynamic culture conditions by utilizing a flow microfluidic chip. This method not only reproduces the physiological accumulation of metabolites but also generates mechanical forces that are instrumental in appropriate genetic expression and cell function.^[^ ^123^ ^]^ Some of these models included microchannels that can be used to provide a vasculature‐like structure within a bigger compartment. This approach was used to create a heart‐on‐a‐chip where the endothelial compartments, supported by a smooth muscle cell compartment, improved the barrier function of the endothelial compartment, an essential property of the endothelium. This is a feature that is often disregarded by other models where endothelial structures lose their integrity due to the lack of support cell types.^[^ ^124^ ^]^

Typically, microfluidics uses channels to perfuse different solutions containing chemicals, thus allowing the generation of chemical gradients that can convert into differentiated interfaces.^[^ ^125^ ^]^ However, this is not limited to chemical gradients, as seen in other works where the channels are used as a means of generating shear stress and mechanical cues. For instance, a lung functional chip took into account both epithelium and endothelium membranes and used air channels to create mechanical features that were central to its function.^[^ ^126^ ^]^ Another lung‐on‐a‐chip also used coupled, vascular and mechanical channels, further allowing effective production of lung epithelial monolayers.^[^ ^127^ ^]^ In this work, an additional membrane coated with gut epithelium cells was used to create a gut‐on‐a‐chip. The model included a vacuum mediated mechanical feature to create a peristaltic‐like mechanical stimulation to bring it closer to the native gut features.^[^ ^128^ ^]^ All these models increase the physiological relevance of the engineered models by recapitulating essential features, i.e., mechanical stimulation and compartmentalization. However, a closer representation of the chosen native tissues could be achieved by including other ECM cues that modulate mechanical properties, providing chemical factors or by embedding the different compartments in a 3D ECM‐like environment that matches the composition to that of the target tissue.

In microfluidics, due to all the compartments being clearly separated, hydrogels can be incorporated in one channel, while a purely liquid media‐based phase can be in the adjacent channel. One of these chips was used to study vascularity in bone marrow patients of hematological malignancies in leukemia. In it, two channels were loaded with cells, one with leukemic and bone marrow cells, and the other with endothelial cells separated by a collagen gel channel. Due to the signaling from the leukemic channel, endothelial cells were observed sprouting toward them.^[^ ^129^, ^130^ ^]^ Similar systems were used to model the blood‐brain barrier (BBB) composed of four compartments, two for media supply (650 µm width), one containing a fibrin‐fibroblasts and another containing a fibrin‐astrocytes (both 800 µm width). After one day, pericytes and human brain microvascular endothelial cells (HBMEC) were added at the border of the fibrin‐astrocyte gel. After one week, the HBMEC displayed newly formed sprouted vessels that reached the fibrin‐fibroblasts compartment. While the pericytes and astrocytes improved the vascular morphology and barrier function, the fibroblast compartment was instrumental in the generation of proangiogenic factor gradients that ultimately led to the model appropriately mimicking key features of the BBB such as narrow vascular morphology, high tight junction expression, and low permeability. The model could further be used as a tool for evaluating drug treatments that involve the central nervous system (CNS) and targeting brain tumor‐associated pathological angiogenesis.^[^ ^131^ ^]^ A pancreatic model with two juxtaposed pancreatic duct adenocarcinoma cells (PDAC) and perfusable endothelial lumens was used to elucidate on PDAC–vascular interactions. With this chip they were able to not only mimic pancreatic ductal cancer invasion and replacement into the blood vessel, but also the effect of a gold‐standard drug, follistatin, on disrupting this process.^[^ ^132^ ^]^ In other models, the heterogeneous architecture was achieved within the same compartment. Ma et al. created a biomimetic liver lobule‐like microtissue that contained a hepatic cord‐like network together with a hepatic sinusoid‐like network.^[^ ^133^ ^]^

Research also found evidence to suggest that in some cases compartments and channels do not need to be in the microscale. In this case, a closed‐circuit to study skeletal metastasis from prostate cancer was created by connecting two wells to a peristaltic pump. Prostate cancer cells positioned in the first well‐travelled to populate the bone stromal component in the second well, displaying key features in metastasis models such as chemotaxis, enabling seeding at targeted tissue sites and improved cancer cell growth at the metastatic site.^[^ ^134^ ^]^

In addition, several of these organ‐on‐a‐chip were found to have the ability to couple to create a system‐on‐a‐chip or even human‐on‐a‐chip model.^[^ ^135^, ^136^, ^137^, ^138^, ^139^ ^]^ For instance, a gastrointestinal tract‐on‐a‐chip and a liver‐on‐a‐chip were coupled to study the effect of nanoparticles over liver injury.^[^ ^140^ ^]^ In this work, a gut chip, a liver chip, a kidney chip, and an arteriovenous reservoir were connected to obtain a multiorgan chip. Each of the chips simultaneously provided several compartments in order to mimic their native composition and functions. This setup provided a platform to study first‐pass drug absorption, distribution, metabolism, toxicity, and excretion in humans.^[^ ^137^ ^]^ In another model, created to test and predict substance effect, compartments were also connected through small channels. The model included several compartments that mimic several organs including brain, bone marrow, and liver, connected to real‐time sensors for in situ monitoring of biophysical and biochemical parameters.^[^ ^138^ ^]^

In conclusion, microfluidics has proven to be an increasingly valuable technology, both by the possibilities it provides in terms of compartmentalization, but also because of the ability to dynamically control complex sets of mechanical and chemical cues. Microfluidics grants precise control over dimensions and provides high reproducibility over each compartment of a model. The ability to include 3D ECM‐like environments, isolate culture conditions of specific compartments and most importantly to allow different types of interactions and set ups between compartments are key advantages of this technique in regards to compartmentalization. With regards to the control it grants over culture conditions, microfluidics is excellent at offering different conditions, such as cyclic stretching in endothelial or pulmonary models, or cyclic shear stress in endothelial or bone models. Moreover, it provides dynamic control over the supply of important nutrients and chemical factors, such as growth factors or chemokines. Despite all its strengths, microfluidics is limited in complexity, and the models are limited to microfluidic chip constraints, i.e., the presence of compartments is inherent to the microfluidic chip and a whole multicompartment structure cannot be retrieved from the chip for further analysis or application. While microfluidics has helped to increase our understanding of how all these features are involved in different biological processes and mechanisms, both in healthy and diseased conditions, it is severely limited in terms of the scale it can reach (microscale), and the type of heterogeneity that can be mimicked structurally. For instance, microfluidics does not allow modeling of higher organ‐scale of full thickness tissues, and while it does support the creating of channel‐based models, it does not allow the study of these structures when they have complex 3D architectures embedded in hydrogel compartments. In reference to the previous section of this review, microfluidics can be used in any configuration of non‐ECM indirect cell contact, but also in gel–gel multicompartment approaches.

### 3.3 3D Bioprinting

3D bioprinting is a technique that has seen a rapid increase in relevance and attention in recent years. It combines additive manufacturing, in which a 3D object is built in a layer‐by‐layer manner combined with tissue engineering, providing promise for advancements through uniting engineering and biological principles to obtain engineered tissues. In this process, 3D printed objects are produced following predesigned architectures that are designed using computer aided software (CAD). CAD helps to obtain consistent, specifically designed and reproducible constructs.^[^ ^141^ ^]^ 3D bioprinting has already been used to produce a wide range of artificial organs including urethras, tracheas, and skin.^[^ ^142^ ^]^ While some of these bioprinted constructs have been used as implantable devices, others make more use of these in drug development and pharmacology.^[^ ^143^ ^]^

**Figure 5:**
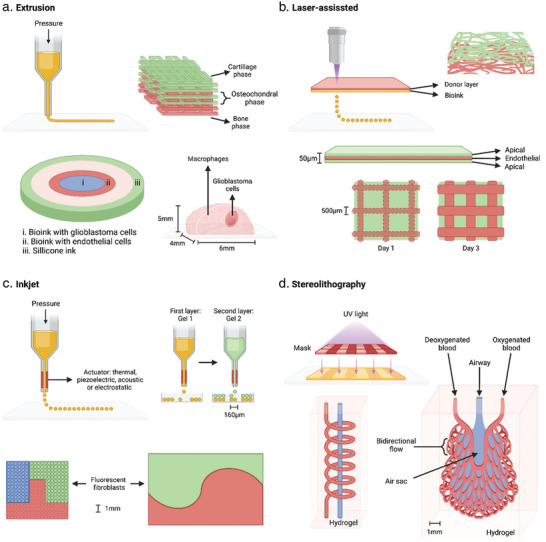
3D Bioprinting modalities and multicompartment examples. a) Extrusion bioprinting: illustrated representation of a hydrogel constructs of osteochondral tissues,^[^ ^32^ ^]^ a minibrain with inner glioblastoma compartment surrounded by macrophages,^[^ ^31^ ^]^ and an ex vivo glioblastoma‐on‐a‐chip model.^[^ ^49^ ^]^ b) Laser‐assisted bioprinting: depiction of a confocal reconstruction of different layers of apical (green) and endothelial cells (red) and a cross‐section illustrating different layering of constructs. An example of horizontal patterning at different time points containing patterned apical (pore) and endothelial cells (grid).^[^ ^176^ ^]^ c) Inkjet bioprinting: diagram showing process for horizontal patterning of a 2‐cell 3D microtissue array,^[^ ^177^ ^]^ and examples of this patterning technique using green/red/blue labeled cells.^[^ ^178^ ^]^ d) Stereolithography bioprinting: representation of a perfusable network model within hydrogel and a complex lung alveoli model containing air sac and oxygenating vascular network.^[^ ^168^ ^]^ Figure made with BioRender.

At the time of this review, there are four main bioprinting modalities in use: extrusion based, inkjet, laser‐induced, and stereolithography bioprinting^[^ ^144^, ^145^ ^]^ (**Figure** **5**). Unlike other additive manufacturing approaches using ceramics, metals or resins, 3D bioprinting uses hydrogels and cells, that can be combined resulting in cell‐laden material as the printing component (Figure 4).^[^ ^141^, ^146^ ^]^ These modalities all share a common feature: the use of cell‐laden hydrogels as printing materials. This is crucial, as the use of cell‐laden hydrogels as the printing component allows for the combination of polymers, ECM materials, and chemical factors to provide the entire range of cues present in native cell microenvironments (**Table** **1**). It should be noted that these gel components can also be combined with thermoplastics or ceramics to provide enhanced structural features if required to or to better mimic mechanical requirements.^[^ ^47^, ^147^, ^148^ ^]^ In addition, one of the main strengths of 3D bioprinting is its ability of producing freeform fabrication. Moreover, 3D bioprinters usually have different printheads or configurations whereby different bioinks can be used simultaneously, granting the possibility of combining multiple printing material compositions.

**Table 1:** Comparison of 3D bioprinting modalities, bioinks, models, and key findings

Bioinks	Cells	Architecture	Key findings	Refs.
Extrusion				
Alginate–gelatin	Aortic valve leaflet interstitial cells (VICs) and smooth muscle cells (SMCs)	Heart valve conduit: 2 compartments laden with each cell type, construct based off CT images.	>80% viability after 7 days. Phenotypic retention was seen for both cell types.	[152]
Alginate–gelatin	Fibroblasts (HMFs), cancer cells, HUVECs	Cancerous tumor construct: cell laden stromal compartment printed with a fully encased cancerous core.	Dynamic cell–cell interaction was seen between compartments. Native stromal characteristics were seen in the stromal compartment.	[153]
BdECM, silicone	GBM cells, HUVECS, cancer cells	Glioblastoma on a chip: 2 concentric compartments surrounded by a silicone chamber wall.	>90%viability Spatial cell organization was seen to closely mimic natural cancer progression	[49]
Esophagus‐derived dECM, PCL	Esophageal cells	Esophageal stent: cell laden reservoir created within PCL stent frame.	>90% viability after 14 days Increase structural stability of stent due to fabrication process	[55]
Fibrinogen based	Keratinocytes, melanocytes, fibroblasts, dermal microvascular endothelial cells, follicle dermal papilla cells, and adipocytes	Full thickness skin construct for wound healing: trilayer skin graft construct.	Wounds were shown to close fully after 3 weeks. Formation of an epidermal barrier like that of native skin.	[8]
Fibrinogen–gelatin (hyaluronic acid), PCL, and PLCL	Urothelial cells (UCs) and Smooth muscle cells (SMCs)	Urethra construct: columnar and spiral tubes with 2 concentric cell laden compartments.	78.3% ± 4.7% UC viability after 7 days. 84.5% ± 5.4% SMC viability after 7 days. Porous scaffold structure allowed for contacts between compartments.	[98]
Fibrinogen‐Gelatin (hyaluronic acid), PCL and PU	Fibroblasts and myoblasts	Muscle‐tendon unit: overlapping rectangular construct with cell laden gel filled gaps.	92.7% ± 2.5% myoblast viability after 7 days. 89.1% ± 3.3% fibroblast viability after 7 days. Elastic muscle side and stiffer tendon side, intermediate characteristic in the middle overlapping interface section. Highly aligned cell morphologies seen.	[99]
Gellan gum	MSCs and HUVECs	Multicompartment osteogenic construct: logpile structure comprising of pairs of different sized compartments.	Viability and osteogenesis were enhanced when crosslinking with strontium.	[101]
GelMA	Endothelial cells and fibroblasts	3D thrombosis on a chip: a sacrificial printed bifurcated channel mold filled with the cell laden hydrogel.	>80% viability after 7 days. Potential for fibroblasts to migrate into blood clots seen.	[154]
GelMA–gelatin	Glioblastoma‐associated macrophages, glioblastoma cells	Minibrain glioblastoma construct: 2 compartments consisting of a hollow printed macrophage wall filled with a glioblastoma laden core.	Cell viability maintained after 10 days. High metabolic activity was seen. Glioblastoma cells recruited and polarized macrophages into a glioblastoma‐assisted specific phenotype.	[31]
Omentum	Endothelial cells, cardiomyocytes	Vascularized heart construct: 2 compartments, comprised of cell laden omentum hydrogel inside an alginate‐xanthan gum support material.	Enhanced cell viability. Anatomically correct cellularized and vascularized heart.	[149]
Inkjet				
Collagen	Type I and II alveolar cells, lung fibroblasts, and lung microvascular endothelial cells	3D alveolar barrier construct: trilayer construct created by printing an endothelial layer onto a porous membrane, a layer of fibroblast laden collagen and collagen ink, and a final layer of alveolar cells.	>90% viability after printing with no significant cell death after 7 days. Enhanced barrier integrity and intercellular junction protein gene expression.	[155]
GelMA–PEGDMA	Myoblasts and tenocytes	Muscle–tendon model: dumbbell‐shaped structure with 0.3 mm gaps, tenocyte laden hydrogel printed around outer posts, myoblast laden hydrogel between the posts.	>95% viability after printing. A continuous tissue construct was formed at the interface between the 2 gels	[156]
starPEG and starPEG‐heparin	MSCs	Layered multicomponent construct: 2 compartments printed in an alternating layer by layer construct.	>80% viability after 7 days. Chemotactic molecular gradients were produced.	[157]
Fibrinogen, PCL	Articular chondrocytes	Cartilage tissue construct: circular construct comprised of alternating PCL and cell laden hydrogel layers.	Viability was >80% after 7 days. Cells proliferated and retained their biological properties.	[158]
PEGDMA	Chondrocytes	Cartilage repair construct: cell laden hydrogel filled defect in an osteochondral allograft plug construct.	89.2 ± 3.6% viability after 1 day with simultaneous photopolymerization. 63.2 ± 9.0% viability after 1 day with continuous photopolymerization after printing. Even distribution of chondrocytes after simultaneous photopolymerization.	[159]
PEGDMA with bioactive glass (BG) and hydroxyapatite (HP) nanoparticles	hMSCs	Osteogenic construct: cylindrical scaffold, coprinted cell‐seeded hydrogel with bioactive ceramic nanoparticles.	Highest viability when HA nanoparticles present, 86.6% ± 6.0% after 21 days. Osteogenic differentiation stimulated significantly when HA present.	[160]
Laser‐induced				
Alginate and EDTA (plasma), collagen	Fibroblasts, keratinocytes	3D multicellular skin construct: bilayered constructs of fibroblast and keratinocyte laden collagen.	No intermixing of keratinocytes and fibroblasts after 10 days. Adheren junctions formed between the cells and gap junctions.	[161]
Cell pellet suspension	HUVECs and MSCs	Cardiac patch construct: adjacent orthogonal grid patterns of HUVECs and MSCs printed onto an elastomer circular patch.	Vessel formation aligning with the grid geometry was seen.	[162]
Collagen	Fibroblasts, keratinocytes	3D multicellular skin construct: stratified skin tissue construct comprised of layering fibroblast laden gel on top of keratinocyte laden gel	Differentiation of keratinocytes in the suprabasal layers was suggested after 11 days. Tissue comprising of fibroblasts producing collagen.	[163]
Collagen	hESC‐LESCs (human embryonic stem cell derived limbal epithelial stem cells) and hASCs	Corneal tissue construct: a rectangular construct consisting of acellular layers printed on top of hASC laden hydrogel layers.	Good viability after printing. Horizontal alignment of hASCs. Attachment to host tissue after 7 days.	[164]
GelMA	Acinar and ductal cells	3D pancreatic cell spheroid: 10 × 10 cells spheroid array.	>90% viability 24 h after printing. Enhanced control over even distribution and the number of cells per spheroid.	[165]
Stereolithography				
PEGDA	Fibroblasts	Microchanneled patch neovasculature construct: PEGDA blocks with100 µm diameter channels.	Significantly enhanced concentrations of secreted VEGF	[166]
PEGDA	ADSCs	Porous scaffold construct: rectangular construct with voids forming micropores.	>90% viability after 7 days. Enhanced cellular viability and activity.	[167]
PEGDA	MSCs and HUVECs	Intravascular alveolar construct: branched concave and convex region structures resembling air sacs with interconnecting airways.	Enhanced blood mixing, flow bidirectionality, and oxygenation of surrounding blood.	[168]
PEGDA and GelMA	Fibroblasts and HUVECs	ECM topography construct: chips designed with patterned. Fibroblasts were seeded onto PEGDA microwell constructs and HUVECs were seeded onto GelMA microwell constructs.	Fibroblasts formed complex multicellular structures aligning with the walls of microwells. HUVECs aligned with the lower hemisphere circumferences of the microwells.	[169]
GelMA	HUVECs	Porous multilayer scaffold: logpile and hexagonal constructs creating rectangular and hexagonal internal pores respectively.	Uniform cell distribution and high cell density. Different pore geometries allowed for selective mechanical properties.	[170]
GelMA and GMHA	Hepatic progenitor cells, HUVECs and ADSCs	Triculture hepatic model: HUVECs and ADSCs in GelMA–GMHA printed on top of hexagonal layers of hepatic cells in GelMA	65% viability after 7 days. Cell reorganization within the maintained hexagonal structure. Enhanced functional and phenotypic properties of hepatic tissue.	[114]

Another key feature is that free‐form fabrication coupled with the ability to use multiple materials in the same construct, allows for the obtention of highly complex architectures. Within the constraints imposed by the technology, 3D bioprinting provides the platform for virtually any structure to be printed. Scientists have even printed an anatomically correct chicken‐sized heart, including its cavities and vessels.^[^ ^149^ ^]^ It is important to consider that the type of construct that can be fabricated is limited by the bioprinting modality that is used.

While other techniques are suitable only for in vitro models, 3D bioprinting could be used in either implants or in vitro models, showing suitable qualities that make it an ideal candidate for both applications. In fact, multicompartment heart valves have been created using an alginate/gelatin bioink, including aortic valve root cells for the walls and leaflet cells for the valve leaflets. The constructs showed good viability, spreading, and phenotype retention, demonstrating the robustness of multimaterial approaches.^[^ ^150^ ^]^ Heart tissue replacements were also engineered with multicompartment alginate–poly(ethylene glycol) (PEG)–fibrinogen HUVEC–iPS cardiomyocytes vertically arranged constructs. This arrangement allowed for cardiomyocyte–HUVEC layers that improved layering in the host, while keeping a highly precise and repeatable compartmentalization of cell types. In addition, they showed the usually overlooked importance of optimizing intercompartment distance for effective paracrine signaling, as the model showed improved behavior in constructs while maintaining a shorter distance between layers.^[^ ^151^ ^]^

Furthermore, 3D bioprinting is also compatible with some of the other techniques, providing opportunities to develop technologies further, i.e., 3D bioprinted constructs coupled with microfluidics chips and flow‐based bioreactors. This provides the possibility of coupling a 3D complex, spatially defined, reproducible structure with automated culture systems in which chemical gradients and different culture conditions can be generated. For example, by incorporating 3D bioprinted constructs, microfluidics systems can be used to engineer systems closely mimicking native tissues, by providing control over dynamic culture conditions both in terms of chemical cues or by generating stresses that emulate mechanical cues, such as respiration or blood flow. Several of these systems could also be combined, just as in microfluidics, to obtain a higher‐scale representation of the chosen tissues or organs, i.e., combining a gut construct in a particular chip or bioreactor with a brain construct in a different chip in order to study gut–brain interactions.

#### 3.3.1 Extrusion‐Based Bioprinting

Extrusion‐based modalities (Figure 5a) use a pneumatic or mechanical force to extrude the material through a nozzle, with the ability to print continuous elements and higher cell densities compared to other modalities, and to print large cell conformations such as spheroids. This is currently the most versatile modality due to the control it offers over extrusion speed, pressure (up to several 100 KPa), temperature, its ability to control material deposition, scalability, and its capability to use different types of bioinks. While it does allow for the printing of a wide range of viscosities, from 30 mPa s^−1^ to 6 × 10^7^ mPa s^−1^, the resolution is typically lower than that of other modalities, usually in the order of 100 µm minimum. Generally, extrusion‐based bioprinters have a lower cost compared to other modalities such as laser‐assisted bioprinting, although some of these printers integrated with cell culture cabinets can have a significantly increased price. Different hydrogels can also be combined in the same construct simultaneously using different printheads some of these printers have. Additionally, many extrusion‐based bioprinters also incorporate other thermoplastic or ceramic printheads allowing them to combine a wider set of materials within the same construct. Printing pressures used in this modality can impair cell viability as demonstrated by the reported values ranging from 40% to 90%, depending on the printing conditions and materials used. The kind of structures that can be printed is also limited by the need to rely on extra support for hanging structures, despite this, extrusion‐based bioprinting is a highly versatile modality that has been used in a wide range of applications, such as bone, cartilage, heart, or cancer^[^ ^13^ ^]^ and 3D bioprinting parameters.

Extrusion‐based bioprinting was used to develop a 2 mm × 2 mm cell‐only construct including a stromal compartment enclosing a cancer compartment. This model was used to understand tumor–stroma interactions, while overcoming the simplicity of some existing 2D models where the architecture and 3D conformation of the biological system is not fully considered. Another tumor–stroma model was created enclosing a breast cancer nodule inside a fibroblast/adipocyte/endothelial cell gel acting as the stromal component. This system helped to enhance the understanding of the effect of several molecules in a context with an increased similarity to the in vivo microenvironment.^[^ ^171^ ^]^ The interaction between breast cancer cells and the surrounding adipose tissue was also studied in a model where a first breast cancer compartment was enclosed by an adipocyte region. This model showed an increased resistance when compared to cancer‐only models. The model replicated in vivo responses and provided a system to better understand breast cancer biology.^[^ ^172^ ^]^ Similarly, a minibrain glioblastoma model, in which glioblastoma‐associate macrophages (GAM) enclosed a compartment of glioblastoma (GBM) cells, was created. This model granted the possibility of studying the close interaction between GAM–GBM. By using the minibrains, the authors proved a crosstalk in which GBM recruits and polarizes macrophages into GAM phenotypes in the presence of GBM, which induced progression and invasiveness of GBM. They also showed how therapeutics can inhibit this interaction, leading to reduced tumor growth and increased sensitivity to chemotherapy.^[^ ^31^ ^]^

#### 3.3.2 Inkjet Bioprinting

Inkjet bioprinting (Figure 5c) was the first additive manufacturing modality developed by modifying commercial inkjet printers to include a chamber and elevator in the *z*‐axis. It uses piezoelectric, thermal, acoustic, or electrostatic actuators to create the droplets and deposits them upon the collecting surface. This modality is very efficient, has a high throughput and is capable of generating low size droplets with a resolution of below 10 µm (minimum print size). However, the level of the accuracy droplet deposition has proven a challenge, making it difficult to create large scale constructs. Inkjet bioprinting is only able to work with low density bioinks below 10^6^ cells mL^−1^ and very low printing viscosities, ≈3, 5, to 12 mPa s^−1^. In addition, cell viability can be affected by frequencies employed by piezoelectrics or high temperatures used by thermal actuators, staying usually ≈85%.^[^ ^160^, ^173^, ^174^, ^175^ ^]^ This modality is not only scalable, but also has a lower price to that of other type of printers, in fact, several systems that were used at the beginning of bioprinting studies were adapted using cheap conventional ink printers.

Inkjet bioprinting was used to fabricate several multicompartment, vertically layered constructs. In a first example, a trilayer construct was fabricated by printing a first layer of endothelial cells to which subsequent layers of fibroblasts and alveolar cells (type I and II) were added. All layers were made of cell‐laden collagen bioinks and between each layer, the constructs were incubated for 24 h. The model exhibited not only viability of over 90% after 7 days, but also showed closer biomimicry of lung tissue structure and function than conventional non‐structured constructs. In particular, the trilayer model displayed higher barrier integrity and intercellular junction protein and gene expression. However, the construct did not consider other cues present in bone such as cyclic mechanical cues associated with aspiration or the presence of an air‐alveolar layer interphase which is present in lung alveoli. Another layered model using MSCs was engineered by printing alternating layers of peg‐based gels in which one of the layer types was functionalized with a heparin region. This printing method had a resolution of 50 µm and allowed for mechanical tailoring of different regions within the construct. Additional inclusion of platelet derived growth factor (PDGF) to the heparin containing layers promoted MSC migration from the non‐PDGF layer toward the growth factor including layer. Although the model does not focus on a particular disease, it provides a platform in which migration or guided morphogenesis can be studied.

#### 3.3.3 Laser‐Assisted Bioprinting

Laser‐induced inkjet printing (Figure 5b) creates droplets by applying a focused laser beam onto a laser energy‐absorbing material donor layer, which combines the donor material and the metallic ribbon structure, resulting in droplets being created on a receiving substrate. This happens because the high pressure generated by the laser upon contacting the metallic ribbon present in the donor layer produces a bubble at the bioink that then drops onto the receiving substrate. The range of supported viscosities is below 300 mPa s^−1^ and the cell densities that can be worked with is below 10^8^ cells mL^−1^, although it provides high cell viability, often over 95%. This technique, due to its nonreliance on a nozzle, can use a wide set of cell‐free and cell‐laden materials including ceramics and hydrogels as bioprinting material. Despite the wide range in resolution (pico‐ to microscales) compared to other modalities, the cost is the highest of all modalities, the throughput is smaller, scalability is lower, and the process of producing the donor layer is costly and time consuming.^[^ ^179^, ^180^, ^181^, ^182^, ^183^ ^]^

Using laser‐assisted bioprinting, multicompartments were achieved by patterning different materials containing different properties and cell types both horizontally and vertically. For instance, a 500 µm thick multicellular skin construct, mimicking the dermis and epidermis, was fabricated by printing a first compartment (consisting of 20 layers) that contained a fibroblast‐laden collagen gel, which was followed by a second compartment (of another 20 layers) made up from a keratinocyte‐laden collagen gel. Cells within each compartment stayed contained, formed both adherens and gap junctions and a basal lamina between dermis and epidermis, following native skin morphogenesis. This type of approach demonstrates the future potential for an in situ skin graft fabrication therapy for patients where large, deep skin defects have occurred. Similarly, a construct made up of 20 layers of keratinocyte‐laden collagen gel followed by 20 layers of fibroblast‐laden gel was found to form a tissue comprised of collagen producing fibroblasts. Another example used different grid structured compartments of MSCs and HUVECs that allowed control over the distance between MSC and HUVEC regions. The construct displayed vessel formation in alignment with the grid, with high HUVEC interconnectivity, whereas no vascular structure was found in randomly seeded monocompartment constructs.

#### 3.3.4 Stereolithography Bioprinting

In stereolithography (SL) (Figure 5d), light‐mediated polymerization builds the 3D object by selectively polymerizing specific regions in each layer inside a pool of materials. It is, therefore, limited to bioinks that consist of photoactivated polymers. This is different to the extrusion‐based modalities as it permits the production of hanging structures without the need of any additional support, making it ideal in producing a wide range of tissue mimetic scaffolds that recapitulate hollow cavities and channels.^[^ ^168^ ^]^ However, the viability can be greatly affected by the amount of photoinitiator or the hydrogel properties needed for it to work with enough consistency and precision, although in recent works researchers have managed to maintain viability ≈85%.^[^ ^184^ ^]^ SL bioprinting overcomes the nozzle‐clogging issue by acting directly onto a layer of material, with no restrictions over the viscosity of the material. Despite this, maximum cell densities lie ≈10^6^ cells mL^−1^ due to the impediment of appropriate crosslinking with higher numbers of cells. An additional disadvantage of this modality is the exposure to ultraviolet (UV) rays required to crosslink the bioinks, which has been shown to produce deoxyribonuclic acid (DNA) damage resulting in long‐term effects that might affect the end results of constructs and models. In addition to these main bioprinting modalities, although still in a more infant state than the abovementioned, volumetric bioprinting uses a combination of SLB and computer tomography framework. This modality works by irradiating light into a photocurable hydrogel 360° around the vertical axis. This allows for the synthesis of highly complex 3D models with challenging hanging structures to be printed in under 1 min, which is impressive considering that other modalities take much longer than this, sometimes even lacking the ability to fabricate the design at all.^[^ ^185^, ^186^ ^]^

Some recent works used stereolithography‐based bioprinting to produce several cavity/channel based architectures. For example, an intravascular alveolar construct was made by creating concave and convex regioned structures resembling air sacs with interconnecting airways and vascular channels to form a branched alveolar‐vascular network. The construct formed a ventilated structure in which enhanced blood mixing, flow bidirectionality, and surrounding blood oxygenation was observed.

## 4 Bioprinting Highlights

Despite all the major developments that have been described already in this review, it is worth highlighting a few key pieces of research that offer promising innovations in biomimetic architectures for the future of multicompartment construct manufacturing using 3D bioprinting. These examples do not necessarily account for the most complex models in terms of compartmentalization, but they instead constitute three major advancements that have occurred in the field in recent years which serve as inspiration to develop better and more accurate biological models, in vitro tools and implants following a compartmentalization framework.

**Figure 6:**
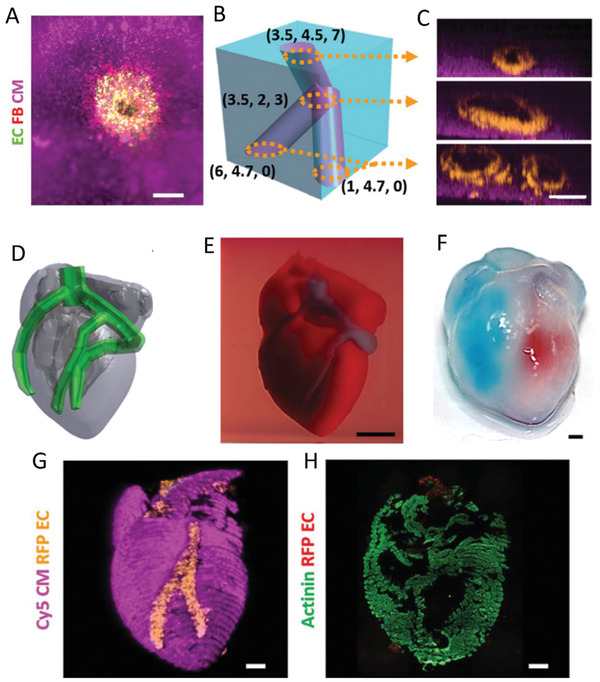
Multicompartment printing of the human brain. a) Cross‐section top view of an endothelial cell vessel (CD31; green) surrounded by fibroblasts (red) within cardiac tissue compartment with cardiomyocytes (actinin; pink). b) Diagram showing bifurcating blood vessel within engineered cardiac tissue (coordinates in mm), and c) the lumens sections of each indicated region of the printed construct. d) Human heart CAD model showing cardiac muscle (gray) and vessel (green) regions. e) A printed heart within a support bath. f) Obtained printed heart after removing of support bath, with left and right ventricles injected with red and blue dyes to demonstrate separated hollow chambers. g) 3D confocal image of the printed human heart (cardiomyocytes in pink, endothelial cells in orange). h) Immunostained cross‐sections of the printed heart showing cardiac (actinin; green) and endothelial compartment (red). Scale bars: (a,c,g,h) = 1 mm, (e) = 0.5 cm, (f) = 50 µm. Adapted with permission under the terms of the CC‐BY license.^[^ ^188^ ^]^ Copyright 2019, the Authors. Published by Wiley‐VCH GmbH.

The first example, corresponding to work done by Lee et al., uses techniques that have been available for a longer time, illustrating how ingenuity and clever engineering with available tools result in world‐class breakthroughs. In this work, a set of shell‐filling bioinks were used to accurately reproduce a full‐size heart, consisting not only of all the cavities but also complex vascular trees throughout the surrounding walls. Even though this work did not support the creation of a fully functional construct, its ability to produce functioning heart valves or ventricle models that mirrored contractability of the heart‐like tissue offers great promise.^[^ ^187^ ^]^ Similarly, Noor et al. were able to print thick perfusable cardiac patches with compartments containing both cardiac tissue and vascular structures. They did this by printing within a support bath (FRESH) that prevented the material from leaking laterally while at the same time providing a support for cavities and hanging structures, while simultaneously crosslinking the printed construct. Following this approach, they were able to not only produce vascularized cardiac constructs of different vascular formations within the heart, like straight vessels or bifurcations, but also print a heart the size of a chicken's heart that contained two separated cavities, mimicking the ventricles with a vascular structure running through it (**Figure** **6**).^[^ ^188^ ^]^

However, reproducing an organ is not necessarily limited to fabricating a full‐size structure but rather the ability to fabricate small building blocks that, once placed together, can display the function of the whole organ one aims to replicate, e.g., nephron building blocks that could show the function of a kidney. Following this rationale, Lewis and co‐workers were able to manufacture vascularized high‐cell density organ building blocks (OBBs). These OBBs were obtained using gelatin‐based sacrificial inks (that would dissolve in culture conditions) to create vascular channels within cell/spheroid‐only constructs. It is important to realize that before full organ engineering is achievable, it is possible that function may be emulated via coupling of functional building blocks. In the future a higher order architecture may arise from this coupling and organs may be formed.^[^ ^189^, ^190^ ^]^

The newest bioprinting technology which uses selective UV shining for crosslinking specific areas of hydrogel solutions, was developed in this work. Although biocompatible hydrogels were used, advancements need to be done in order to use this technique with living cells and to further be able to create compartments within them, other than cavities or vascular structures. With the name “SLATE,” stereolithography apparatus for tissue engineering, Grigoryan et al. developed a stereolithography platform to generate a highly intricate set of toroid structures. These SLATE were ultimately used as models of pulmonary alveoli in which air‐cavities surrounded by vascular networks provided the media to hypothetically transport oxygenated blood into the system. After considering the limitations of current compatible bioinks, they also produced a pancreatic construct with a base containing a set of channels imitating vascular structures. This was supported by a functional pancreatic hydrogel printed via traditional bioprinting principles to offer better biocompatibility to the structure.^[^ ^168^ ^]^ In some recent work following a similar approach, researchers were able to produce perfusable constructs that matched the architecture and mechanical properties of various organs present in the human body.^[^ ^191^ ^]^

## 5 Conclusions

Researchers have been trying to mimic and engineer tissues and organs for decades now, both with implantable and modeling purposes in mind. However, while major advances have been made in the field, some challenges remain unsolved. The main challenge, in our opinion, which we have discussed throughout this review, is the degree of resemblance between engineered and native tissues. This is due to the inherent heterogeneity tissues display, which may have been overlooked so far. However, replicating this heterogeneity in engineered tissues is key to model both complex but also more simple compatible tissues.

Multicompartment models could provide an effective scenario whereby using various approaches and technologies, tissue heterogeneity can be mimicked. Similarly, the “multicompartment prism” phenomenon in tissue engineering has proved to be helpful in designing models and construct layouts that better mimic tissues. These different compartments are essential in obtaining a more complete picture of the different parts interacting on a certain biological system. Given the myriad of possibilities biomaterials provide, native tissue physicochemical properties can be matched.

The complexity of these multicompartment models, can vary dramatically from distinctive and separated layers of several cell types, to 3D bioprinted structures that possess both the closely matched architecture, cellular composition, and ECM composition of native tissues. However, even more simple 2D multicompartment models could overcome oversimplistic homogeneous models.

This review also offered evidence to suggest that while micropatterning and microfluidics technologies facilitate multicompartment constructs, they rarely involve 3D scenarios to provide the cells with the cues present in the ECM. When they do, however, the structural complexity of the engineered tissues and models are usually limited in size and application, being more suited for in vitro models than, for instance, implantable purposes.

3D bioprinting, despite being in a very early development stage, can overcome all those challenges and limitations, permitting the obtention of structures of different configurations, even with conflictive features such as perfusable channels or cavities. Moreover, 3D bioprinting could also be paired with technologies such as microfluidics or perfused systems to receive benefits from the perfusable features it provides while offering better structural mimicking of native tissues. In fact, vascularization, and in general, the presence of channels within engineered tissue constructs remains one of the main challenges to tackle to bridge the gap between native and engineered tissue architecture. This is due to the many roles vessels and the different channels have in tissues and organs, from keeping the balance of certain chemicals, to the supply of blood, oxygen, and other essential metabolites to the different cells within the body. The size limit, fabrication speed, and architectural limitations are readily being pushed forward collectively by the academic and industrial community due to the potential it holds. Perhaps in a couple of decades we will be able to see 3D bioprinted constructs that emulate native complex organs both in shape and function.

In the upcoming future, we think the field will progress in three main directions. The first, to establish and engineer new manufacturing technologies that will offer more control and range in terms of achievable architectures of the produced constructs. The second, the development of novel biomaterials that will be more tailorable, with a more defined chemistry and composition, and that would also allow for higher control over the physicochemical and cell instructive cues they can provide over time. Finally, we see the field focusing on putting together the first two directions, creating a new generation of constructs that include the fabrication of not only architecture/structure mimicking constructs, but also the ability to recapitulate the functionality of the given tissue or organ on a bigger scale.

## Conflict of Interest

The authors declare no conflict of interest.
